# Heritability of Cortisol Production and Metabolism Throughout Adolescence: A Twin Study

**DOI:** 10.1210/clinem/dgz016

**Published:** 2019-10-14

**Authors:** Britt J van Keulen, Conor V Dolan, Ruth Andrew, Brian R Walker, Hilleke E Hulshoff Pol, Dorret I Boomsma, Joost Rotteveel, Martijn J J Finken

**Affiliations:** 1 Emma Children’s Hospital, Amsterdam UMC, Vrije Universiteit Amsterdam, Pediatric Endocrinology, Amsterdam, The Netherlands; 2 Department of Biological Psychology, Vrije Universiteit Amsterdam, The Netherlands; 3 Centre for Cardiovascular Science, University of Edinburgh, Queen’s Medical Research Institute, Edinburgh, UK; 4 Institute of Genetic Medicine, Newcastle University, Newcastle upon Tyne, UK; 5 Department of Psychiatry, Brian Center Rudolf Magnus, University Medical Center Utrecht, Utrecht, The Netherlands

## Abstract

**Context:**

Inter-individual differences in cortisol production and metabolism emerge with age and may be explained by genetic factors.

**Objective:**

To estimate the relative contributions of genetic and environmental factors to inter-individual differences in cortisol production and metabolism throughout adolescence.

**Design:**

Prospective follow-up study of twins.

**Setting:**

Nationwide register.

**Participants:**

218 mono- and dizygotic twins (*N* = 109 pairs) born between 1995 amd 1996, recruited from the Netherlands Twin Register. Cortisol metabolites were determined in 213, 169, and 160 urine samples at the ages of 9, 12, and 17, respectively.

**Main outcome measures:**

The total contribution of genetic factors (broad-sense heritability) and shared and unshared environmental influences to inter-individual differences in cortisol production and activities of 5α-reductase, 5β-reductase, and 11β-hydroxysteroid dehydrogenases and cytochrome P450 3A4.

**Results:**

For cortisol production rate at the ages of 9, 12, and 17, broad-sense heritability was estimated as 42%, 30%, and 0%, respectively, and the remainder of the variance was explained by unshared environmental factors. For cortisol metabolism indices, the following heritability was observed: for the A-ring reductases (5α-and 5β-reductases), broad-sense heritability increased with age (to >50%), while for the other indices (renal 11β-HSD2, global 11β-HSD, and CYP3A4), the contribution of genetic factors was highest (68%, 18%, and 67%, respectively) at age 12.

**Conclusions:**

The contribution of genetic factors to inter-individual differences in cortisol production decreased between 12 and 17y, indicative of a predominant role of individual circumstances. For cortisol metabolism, distinct patterns of genetic and environmental influences were observed, with heritability that either increased with age or peaked at age 12y.

Cortisol, the main product of the hypothalamus–pituitary–adrenal (HPA) axis, is a crucial steroid hormone in the physiological stress response following homeostasis disturbance (1). Dysregulation of HPA axis activity has been associated with cardiovascular diseases and psychiatric conditions including major depressive disorder, posttraumatic stress disorder, panic disorder, and chronic anxiety (2–4). It has been recognized that experiences in early life may induce permanent alterations in the settings of several endocrine systems, including the HPA axis (5, 6).

There is a paucity of data on the magnitude of the contribution of genetic factors to variance in HPA axis activity. Several studies in monozygotic (MZ) and dizygotic (DZ) twins addressed the relative contributions of genetic and environmental factors on serum and salivary cortisol levels. However, the magnitude of the heritability estimates varies (Table 1). A meta-analysis of 5 twin studies published before 2001, of which 4 were conducted in adults and 1 in both children and adults, estimated the heritability of basal cortisol assessed in serum or saliva at 62% (7). The total sample size in the meta-analysis was small (209 MZ and 190 DZ pairs), and power analyses included in the paper indicated that the statistical power to distinguish between genetic and shared environmental influences was low. Later studies reported lower heritability estimates for salivary cortisol levels and showed that those estimates differed at different time points of the circadian cortisol rhythm (8–11). In adults, the heritability of cortisol level was 32% to 34% in samples obtained directly after awakening or 30 minutes post-awakening (9). In the evening samples obtained from these adults, the heritability was found to equal zero. In children, the heritability estimates were 28%, 60%, and 8%, directly after awakening, 30 minutes post-awakening, and in the evenings, respectively (8). In summary, previous twin research consistently found that variation in morning salivary, serum, and hair cortisol levels appears to be at least partially heritable (Table 1). Cortisol secretion varies with age, and inter-individual differences in diurnal cortisol levels are presumed to emerge during the second decade of life (12).

**Table 1.** Summary of Previous Cortisol Heritability Twin Studies

First Author	Publication	Medium	Type	Sample Size (*n* twins)	Sex	Age	Heritability (%)
Bartels^a^ (7)	2003	Saliva and plasma	Sampling across the day	798 (418 MZ, 380 DZ)	Males and females	Children and adults	62
Bartels (10)	2003	Saliva	Awakening, 30–60 minutes postawakening, 12.30 hours, 20.30 hours	360	Males and females	Children (12 years old)	22–24, 56–59, 30-21, 0
Federenko (13)	2004	Saliva and plasma	After stress exposure (3 times, salivary, and total cortisol)	116 (66 MZ, 50 DZ)	Males	Children and adults (16–24 years old)	8, 56, 100, 32, 36, 98
Kupper (9)	2005	Saliva	Awakening, 30 minutes postawakening and evening	471 (199 MZ, 272 DZ)	Males and females	Adults	34, 32, 0
Schreiber (14)	2006	Saliva	Prior to dinner	412 (176 MZ, 236 DZ)	Males and females	Children (8 years old)	0
Ouellet-Morin (15)	2008	Saliva	Cortisol reactivity	346 (130 MZ, 216 DZ)	Males and females	Children (19 months old)	51
Steptoe (16)	2009	Saliva	Cortisol reactivity	150 (80 MZ, 70 DZ)	Males and females	Children (11 years old)	58, 60, 56, 44
Riese (17)	2009	Saliva	Awakening, and 30, 45, and 60 minutes postawakening	250 (184 MZ, 102 DZ)	Females	Adults	46, 69, 69, 52
Franz (18)	2010	Saliva	Awakening and 30 minutes postawakening, 10.00 hours, mean output across the day, mean CAR	401 (196 MZ, 205 DZ)	Males	Adults	56, 48, 42, 43, 64
Gustafsson (8)	2011	Saliva	Awakening, 30 minutes postawakening, bedtime	302 (154 MZ, 148 DZ)	Males and females	Children (9–16 years old)	28, 60, 8
Van Hulle (19)	2012	Saliva	Awakening, slope, evening level	904 (314 MZ, 578 DZ)	Males and females	Children (7–8 years old)	31, 32, 0
Ouellet-Morin (11)	2016	Saliva	Awakening, CAR, diurnal change	592 (280 MZ, 312 DZ)	Males and females	Children (14 years old)	28, 50, 31
Rietschel (20)	2016	Hair cortisol	3 cm hair	58 (16 MZ, 42 DZ)	Males and females	Children and young adults (15 years old)	0
Tucker-Drob (21)	2017	Hair cortisol	3 cm hair	1214 (376 MZ, 838 DZ)	Males and females	Children (8–20 years old)	65
Rietschel (22)	2017	Hair cortisol	3 cm hair	671 (232 MZ, 374 DZ, trizygotic twins)	Males and females	Children (15 years old)	72

^a^Bartels et al (7) included an analysis of 5 original, comparable twin studies focusing specifically on basal cortisol levels (Wust et al (23), Froehlich et al (24), Inglis et al (25), Linkowski et al (26), and Meikle et al (27)).

All studies are published after the meta-analyses in 2003 of Bartels et al (7). The numbers presented in this table were extracted from (7–11, 13–22).

Abbreviations: CAR, cortisol awakening response; DZ, dizygotic; MZ, monozygotic.

Earlier studies have focused on cortisol levels in serum or salivary. These cortisol levels represent the net effect of cortisol production and metabolism. The contributions of genetic and environmental factors to individual differences in cortisol production or metabolism, as determined by cortisol metabolite excretion in urine, have yet to be studied. Day-to-day excretion of cortisol in the urine is moderately stable (28). Cortisol is metabolized to cortisone by 11β-hydroxysteroid dehydrogenase (HSD) type 2 in the kidney, while the reverse reaction occurs by 11β-HSD type 1 in liver and adipose tissue. Cortisol is also metabolized irreversibly by the A-ring reductases (5α-reductase and 5β-reductase), and cytochrome P450 (CYP) 3A4 in the liver. Cortisol metabolism is stable across the menstrual cycle (29). The aim of the current study was to focus on indices of cortisol production and metabolism across adolescence, and to estimate the relative contributions of genetic and environmental factors to cortisol production and metabolism in a sample of children who were registered at birth in the Netherlands Twin Register (NTR). The twins took part in a longitudinal study and were seen at 9, 12, and 17 years of age (30).

## Methods

### Participants

**Figure 1.**
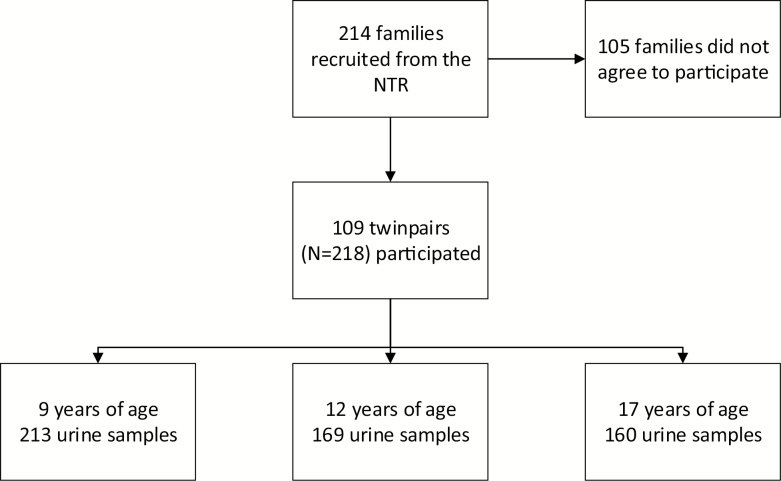
This flowchart presents the enrollment of participants for this study.

We conducted a prospective follow-up study in MZ and DZ twin pairs. Participants in this study were recruited from the NTR (31, 32) and invited to take part in the BrainScale study of cognition, hormones, and brain development (30, 33). Parents of twins born between 1995 and 1996 were invited by letter 4 to 8 weeks before the 9th birthday of the twins. Two weeks later, the parents were approached by phone, to explain about the study and to ask them if they consented to their children taking part. Of the 214 families who were approached, 109 consented to take part (51%). Seventy-eight and seventy-three percent of the participants took part in the follow-up study and provided samples at the ages of 12 and 17 years, respectively (Fig. 1).

Information on demographics, gestational age, birth weight, and other prenatal and perinatal variables was collected by questionnaire at the time of NTR registration (34). Twins were categorized based on zygosity and sex: MZM (MZ males), DZM (DZ males), MZF (MZ females), DZF (DZ females), or DZOS (DZ opposite sex male–female or female–male). To establish zygosity, participants were requested to collect buccal swabs from which deoxyribonucleic acid (DNA) was isolated. All DNA samples were tested for genome-wide single nucleotide polymorphic (SNP) markers (35).

BrainScale is a collaborative project between the NTR at the Vrije Universiteit Amsterdam and University Medical Center Utrecht. The project was approved by the Central Committee on Research Involving Human Subjects of The Netherlands (CCMO), and studies were performed in accordance with the Declaration of Helsinki. Parents signed informed consent forms for the children and for themselves. Children signed their own informed consent forms at the third measurement. Parents were compensated for travel expenses, and children received a present or gift voucher at the end of the testing day. In addition, a summary of cognition scores and a printed image of their T1 brain magnetic scan imaging scan, when available, were provided afterward.

The current study was approved by the medical ethics committee of the Amsterdam UMC, location VUmc.

### Study protocol

At the ages of 9, 12, and 17 years, participants visited the study site for different tests. In the week prior to the study visit, urine samples were collected upon awakening in specially provided tubes. Participants or their parents were requested to store the tubes in their refrigerator and to bring them to the study visit. Samples were subsequently stored at –20° and –80° Celsius and thawed only once just before analysis. Urine samples were available from 47 MZ (23 male, 24 female) and 62 DZ twin pairs (22 male, 21 female, 19 opposite sex).

Participants were instructed to bring any packages of recently used medication to the study visits, revealing that there was hardly any recent use of medication in our sample.

### Laboratory analysis

Analysis of cortisol metabolites was conducted at the Edinburgh Clinical Research Facility Mass Spectrometry Core Laboratory. Glucocorticoid metabolites were measured by gas chromatography-tandem mass spectrometry (GC-MS/MS) (36). Samples were analyzed in 15 batches. Ratios of cortisol metabolites representing the activities of various enzymes involved in cortisol metabolism were calculated, as depicted in Table 2.

**Table 2.** Ratios Indicating Enzyme Activity

Urine Metabolites	Index	9-Year Median (IQR)	12-Year Median (IQR)	17-Year Median (IQR)
(THF + allo-THF + THE + α-cortol + β-cortol + α-cortolone + β-cortolone)/creatinine	Sum of cortisol metabolites (cortisol production rate)	0.5666 (0.29)	0.5374 (0.28)	0.3777 (0.30)
Allo-THF/cortisol	5α-reductase activity	8.2273 (7.55)	6.9109 (6.19)	5.0453 (6.85)
THF/cortisol	5β-reductase activity (a)	8.0941 (7.43)	9.6624 (7.23)	7.8718 (31.42)
THE/cortisone	5β-reductase activity (b)	23.8426 (13.94)	25.1619 (16.93)	24.5388 (17.03)
Cortisol/cortisone	Renal 11β-HSD type 2 activity	0.7290 (0.62)	0.6677 (0.41)	1.1074 (0.66)
(THF + allo-THF)/THE	Global 11β-HSD activities	0.6079 (0.43)	0.5109 (0.36)	0.6176 (0.40)
6-OH cortisol/cortisol	Cytochrome P450 3A4 activity	1.5363 (1.21)	1.5531 (1.44)	1.6866 (1.02)

Abbreviations: IQR, interquartile range; THF, tetrahydrocortisol; THE, tetrahydrocortisone; HSD, hydroxysteroid dehydrogenase.

### Statistical analysis

Outliers, defined as any value greater than 3 standard deviations above the phenotypic mean, were excluded from statistical analysis (on average 6 per index). Twin pairs with highly discordant outcomes, as assessed by visual inspection of scatterplots, were also removed (on average, 0.76 per index), given their impact on the MZ and DZ correlations. We corrected for batch effects by fitting a random effects model to the twin data, which included batch as a random effect (37). As the sampling unit is twin pairs, we included in the model family as a random effect to estimate the MZ and DZ twin (intraclass) correlations. These analyses were done in R 3.4.2. using the nlme library (nonlinear mixed-effects models) (38, 39). The batch-corrected phenotypic data were subject to subsequent analyses, as described in the following text.

**Figure 2.**
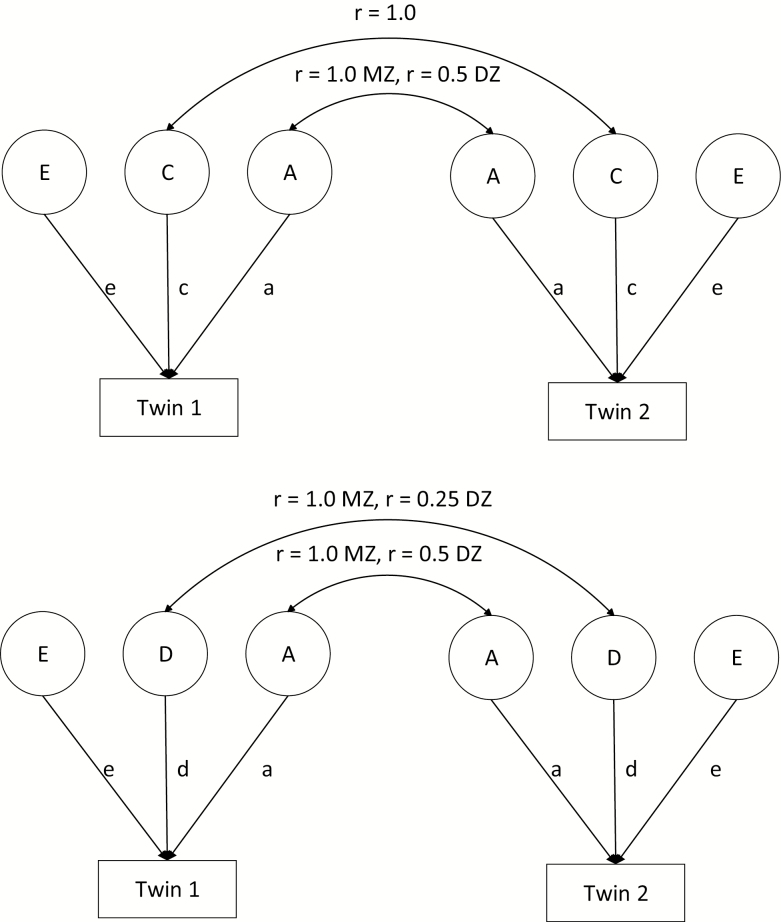
Path diagram representing the ACE (top) and ADE (bottom) model.

The classical twin design exploits the fact that MZ twins share 100% of their alleles identically by descent (IBD; from the biological parents), and DZ twins, on average, share 50% of their alleles IBD (40). This difference in genetic relatedness allows us to estimate the relative contributions of genetic and environmental factors to phenotypic individual differences in terms of genetic and environmental variance components. In practice, either an ACE model or an ADE model is fitted to the twin data, where A stands for additive genetic effects, D for nonadditive or dominance (genetic) effects, C for shared (or common) environmental effects, and E for unshared environmental effects. MZ and DZ pairs differ in their genetic relatedness but share the prenatal and postnatal environment. The extent to which resemblance in both types of twins is not explained by differential genetic resemblance is the basis for identification of common environmental factors. An ACE model is fitted to twin data if the MZ phenotypic correlation (r_MZ_) is smaller than 2 times the DZ correlation (r_DZ_; r_MZ_ < 2*r_DZ_). With 2 groups of relatives (ie, MZ and DZ twins), it is not possible to estimate 4 variance components; that is, a model including nonadditive genetic factors in addition to common environment is not identified. A choice needs to be made to evaluate an ACE or ADE model. If (r_MZ_>2*r_DZ_), an ADE model is chosen. Unshared environmental factors incorporates aspects of the environment that are child-specific and results in differences between twin pairs (ie, the differences within MZ pairs are due to these factors). Unshared environmental factors also includes measurement error. Path diagrams of the ACE and ADE models are shown in Fig. 2. We fitted a series of univariate models.

We used OpenMx to fit the ACE or ADE model to the batch corrected twin data (41). The genetic and environmental variance components were estimated by maximum likelihood. We included as fixed covariates in the model sex and gestational age, since these factors have previously been associated with HPA axis activity (12, 42). Next, we present the estimates of total standardized variance components. The standardized variance components were obtained by dividing by the total variance.

## Results

A total of 218 twins who were born in the Netherlands were enrolled in this study, including 94 MZ and 124 DZ twins. The MZ pairs included 23 MZM twin pairs and 24 MZF twin pairs. The DZ twins included 22 DZM twin pairs, 21 DZF twin pairs, and 19 DOS twin pairs. In total, 213, 167, and 162 samples were analyzed at 9, 12 and 17 years of age, respectively.

Fifty percent (*n* = 109) of all participants was male. Mean (±SD) gestational age was 36.8 weeks (±1.72), and mean (±SD) birthweight was 2602 gram (±475). Participants were tested at 9.1 (±0.1), 12.2 (±0.3), and 17.2 (±0.2) years of age. Mean standard deviation score (Z score) (±SD) body mass index (weight[kg]/height[m]^2^) was 0.14 (± 0.93), 0.45 (± 1.00), and 0.26 (± 1.57) at the ages 9, 12, and 17 years, respectively (43).

The twin correlations and variances for each index are presented in Table 3. The DZ correlations (average 0.257) were lower than the MZ correlations (average 0.415) in 17 of the 21 phenotypes, pointing toward a relatively simple additive genetic model. Thirteen of the phenotypes appeared to be consistent with an ACE model. We note that some correlations were zero (MZ: 5β-reductase activity at age 12; DZ: renal 11β-HSD type 2 activity). We attribute this to sampling fluctuation, given the relatively small sample sizes.

**Table 3.** Intraclass Correlations of Batch Corrected Data with Fixed Covariates Sex and Gestational Age

			MZ		DZ					
Phenotype	Age (years)	*n*	ICC	Variance	ICC	Variance	Model	b0	bsex	bGA
Cortisol production rate	9	167	0.294	0.031	0.037	0.054	ADE	–0.357	–0.014	0.010
	12	147	0.358	0.059	0.021	0.039	ADE	0.142	–0.017	–0.004
	17	148	0.055	0.056	0.307	0.055	ACE	0.523	0.047	–0.015
5α-reductase activity	9	188	0.547	30.448	0.467	25.942	ACE	–24.209	0.075	0.656*
	12	141	0.749	18.898	0.522	18.869	ACE	–2.467	–1.927*	0.094
	17	151	0.489	12.039	0.400	19.728	ACE	–0.180	3.179**	–0.036
5β-reductase activity (a)	9	190	0.466	21.215	0.459	21.320	ACE	–7.148	0.421	0.189
	12	156	0.000	23.388	0.245	35.027	ACE	3.263	0.238	–0.094
	17	153	0.645	13.693	0.062	13.402	ADE	–1.795	1.178	–0.032
5β-reductase activity (b)	9	204	0.691	94.811	0.452	62.492	ACE	11.922	–0.453	–0.312
	12	165	0.548	128.790	0.120	102.764	ADE	2.128	–2.269	–0.028
	17	156	0.565	91.055	0.400	201.990	ACE	–18.345	6.839**	0.444
Renal 11β-HSD type 2	9	195	0.206	0.120	0.000	0.119	ADE	–0.526	0.005	0.014
activity	12	159	0.640	0.056	0.228	0.067	ADE	–1.344	0.064	0.036**
	17	155	0.220	0.154	0.305	0.186	ACE	–1.195	–0.102	0.034
Global 11β-HSD	9	197	0.548	0.048	0.383	0.029	ACE	–1.214	0.038	0.032**
activities	12	162	0.246	0.076	0.043	0.030	ADE	–0.530	0.047	0.014
	17	153	0.123	0.048	0.166	0.056	ACE	–0.514	0.068	0.013
Cytochrome P450 3A4	9	185	0.275	0.686	0.215	0.473	ACE	–1.574	0.066	0.043
activity	12	144	0.626	1.486	0.233	0.628	ADE	1.004	–0.080	–0.026
	17	148	0.427	0.318	0.343	0.670	ACE	1.286	0.078	–0.035

*P < 0.05, ** *P* < 0.01.

Abbreviations: MZ, monozygotic; DZ, dizygotic; ICC, intraclass correlation coefficient; HSD, hydroxysteroid dehydrogenase; b0, intercept; bsex, regression coefficient sex; bGA, regression coefficient gestational age.

The relative contributions of genetic (additive and nonadditive), shared environmental, and unshared environmental factors, along with 95% confidence intervals, are displayed in Table 4. At age 9, the contribution of variation in the cortisol production rate was explained for approximately 40% by genetic factors and 60% by unshared environmental factors. The contribution of genetic factors to the inter-individual differences in cortisol production rate decreased with age. At age 17, genetic factors were no longer contributing to the cortisol production rate. Variation at this age is mainly (for 79%) explained by unshared environmental factors. For indexes representing A-ring reductases activity, the contribution of genetic factors to inter-individual differences was found to increase with age. The contribution of genetic factors to the inter-individual differences in activities of renal 11β-HSD type 2 activity, CYP3A4, and 11β-HSD first increased from 9 to 12 years of age and then decreased from 12 to 17 years.

**Table 4.** Estimates of Genetic (A, D) and Environmental (C, E), Raw and Standardized

Index		Age (Years)	A (Additive Genetic)	C (Shared Environment)	D (Dominance)	E (Unshared Environment)	Broad-Sense Heritability (A+D) (%)
Cortisol production rate	Raw	9	0.000	—	0.019	0.027	—
		12	0.000	—	0.014	0.033	—
		17	0.000	0.012	—	0.044	—
	Standardized	9	0.000 (0.00–0.56)	—	0.415 (0.00–0.69)	0.585 (0.31–1.00)	42
		12	0.000 (0.00–0.51)	—	0.301 (0.00–0.54)	0.699 (0.46–1.00)	30
		17	0.000 (0.00–0.41)	0.210 (0.00–0.42)	—	0.790 (0.57–1.00)	0
5α-reductase activity	Raw	9	4.163	10.568	—	12.827	—
		12	8.562	5.579	—	4.739	—
		17	8.574	1.650	—	6.571	—
	Standardized	9	0.151 (0.00–0.68)	0.384 (0.00–0.63)	—	0.466 (0.29–0.69)	15
		12	0.454 (0.00–0.84)	0.296 (0.00–0.67)	—	0.251 (0.14–0.46)	45
		17	0.511 (0.00–0.78)	0.098 (0.00–0.57)	—	0.391 (0.22–0.72)	51
5β-reductase activity (a)	Raw	9	0.000	9.646	—	11.176	—
		12	0.000	3.236	—	26.233	—
		17	0.000	—	8.612	4.963	—
	Standardized	9	0.000 (0.00–0.50)	0.463 (0.00–0.61)	—	0.537 (0.37–0.72)	0
		12	0.000 (0.00–0.00)	0.110 (0.00–0.32)	—	0.890 (0.67–1.00)	0
		17	0.000 (0.00–0.71)	—	0.634 (0.00–0.78)	0.366 (0.22–0.62)	63
5β-reductase activity (b)	Raw	9	17.713	30.521	—	27.502	—
		12	3.856	—	53.255	55.417	—
		17	90.367	0.000	—	46.988	—
	Standardized	9	0.234 (0.00–0.72)	0.403 (0.00–0.68)	—	0.363 (0.24–0.54)	23
		12	0.034 (0.00–0.66)	—	0.473 (0.00–0.69)	0.493 (0.31–0.76)	51
		17	0.658 (0.14–0.81)	0.000 (0.00–0.31)	—	0.342 (0.19–0.64)	66
Renal 11β- hydroxysteroid dehydrogenase type 2 activity	Raw	9	0.000	—	0.014	0.106	—
		12	0.012	—	0.032	0.021	—
		17	0.000	0.047	—	0.125	—
	Standardized	9	0.000 (0.00–0.27)	—	0.113 (0.00–0.39)	0.887 (0.61–1.00)	11
		12	0.185 (0.00–0.79)	—	0.494 (0.00–0.81)	0.321 (0.19–0.58)	68
		17	0.000 (0.00–0.54)	0.274 (0.00–0.47)	—	0.726 (0.46–0.95)	0
Global 11β- hydroxysteroid dehydrogenase activities	Raw	9	0.000	0.018	—	0.020	—
		12	0.009	—	0.000	0.040	—
		17	0.000	0.008	—	0.044	—
	Standardized	9	0.000 (0.00–0.59)	0.476 (0.00–0.62)	—	0.524 (0.36–0.70)	0
		12	0.183 (0.00–0.39)	—	0.000 (0.00–0.39)	0.817 (0.61–1.00)	18
		17	0.000 (0.00–0.47)	0.151 (0.00–0.36)	—	0.849 (0.53–1.00)	0
Cytochrome P450 3A4 activity	Raw	9	0.233	0.000	—	0.373	—
		12	0.206	—	0.527	0.360	—
		17	0.327	0.000	—	0.211	—
	Standardized	9	0.385 (0.00–0.62)	0.000 (0.00–0.40)	—	0.616 (0.38–0.96)	39
		12	0.188 (0.00–0.81)	—	0.482 (0.00–0.82	0.330 (0.18–0.66)	67
		17	0.608 (0.00–0.78)	0.000 (0.00–0.48)	—	0.393 (0.22–0.75)	61

## Discussion

In this longitudinal study of twin pairs followed between the ages of 9 and 17 years, we demonstrated the relative contributions of genetic and environmental factors to the indices of cortisol production and metabolism throughout adolescence. The most important finding from our study is that the environment plays a key role in the production of cortisol, evidenced by the predominant and increasing contribution with age of unshared environmental factors. In addition, we found distinct patterns of genetic and environmental contribution to the different cortisol-metabolizing pathways.

A previous meta-analysis of twin studies estimated heritability of basal cortisol, representing the net effect of cortisol production and elimination, at 62% (7). Despite this observation, others demonstrated no significant SNP heritability for plasma or salivary morning cortisol (44). The interpretation of such discrepant findings is complicated, when considering that approximately 50% of the variance in salivary cortisol was dependent on day-to-day fluctuations (45).

In our study, cortisol production was mainly determined by unshared environmental factors already at age 9, and the contribution of unshared environmental factors was found to increase with age. This lends support to previous observations suggesting that the settings of the HPA axis are mainly determined by individual circumstances (46). There is overwhelming evidence from animal experiments and epidemiological studies demonstrating that experiences in early life may program future HPA axis activity, with data linking poorer quality of parental care to increased HPA axis activity along with increases in mental illnesses and cardiovascular diseases (47–49). These observations could be attributed to increased DNA methylations status and a reduced expression of the glucocorticoid receptor promotor in hippocampal regions (6, 50, 51). However, more recent evidence suggests that the time window in which epigenetic programming could occur may extend into adulthood (52–54). In male middle-aged twins, saliva cortisol levels showed significant cortisol heritability estimates for laboratory measures, but not for measures in the home situation, suggesting that genetic factors influence cortisol responses to specific environmental stressors (18).

In animal experiments, the influences of early life experiences can be observed by randomly allocating offspring into groups with different amounts of exposure to the factor of interest, whereas in humans determining the long-term impact of life experiences on HPA axis activity is more challenging. Previous research in humans linked early-life experiences like battering, neglect, emotional maltreatment, perinatal malnutrition, low birthweight, and prematurity with future HPA axis activity (48, 55–58). The findings obtained from these studies are inevitably confounded by factors associated with both exposure and outcome, such as low socio-economic class and low household income. Twin studies offer a powerful tool to study environmental contributions by controlling for the family background.

In our study, cortisol metabolism was, in contrast to cortisol production, considerably influenced by genetic constitution, and our findings suggest distinct patterns of genetic and environmental contribution to the different metabolic pathways. The activities of the A-ring reductases are, unlike cortisol production, less influenced by unshared environmental factors, especially later in life. Variation in A-ring reductases activity was for 0% to 23%, 0% to 51% and 51% to 66% explained by (nonadditive and additive) genetic factors at 9, 12, and 17 years of age, respectively, indicating a predominant role of genetic constitution in the regulation of A-ring reductases with age. The influences of genetic constitution on the activities of 11β-HSD isozymes and CYP3A4 increased from 9 to 12 years and then decreased from 12 to 17 years of age. An explanation for the varying influences found for these enzymes may lie in a complex interplay between cortisol metabolizing enzymes and the hormonal regulators of puberty and the pubertal growth spurt, in particular growth hormone and insulin-like growth factor 1. A previous twin study has shown that the pubertal growth spurt, peaking between 12 and 14 years, is strictly genetically regulated (59). In addition, there is strong evidence suggesting that these hormones affect the clearance of cortisol (29, 60, 61).

Our study has several strengths and limitations. The major strength of our study was the long-term follow-up. Furthermore, participants were recruited from a nationwide twin registry, and the numbers lost to follow-up were acceptably low. Consequently, selection bias is unlikely to explain our results. Another strength of our study was the use of GC-MS/MS analysis, providing highly reliable measurements. Thus, the findings as presented are unlikely to be explained by measurement error. However, our study also has its limitations. Participants were requested to collect early-morning urine samples. Preferably, a 24-hour urine sample would have been analyzed, since cortisol is secreted in a circadian rhythm. Next, sampling started no earlier than at age 9, so that there remains a lack of knowledge on the genetic and environmental etiology in early childhood. Moreover, differences between boys and girls were observed, necessitating further research. Additionally, due to limited sample size, resulting in large confidence intervals and some remarkable twin correlations (eg, MZ correlation < DZ correlation for cortisol production rate at age 17), our results have to be interpreted cautiously. Finally, the collected samples were not randomly distributed across the different analytical batches, which may have led to systematic error. More specifically, samples from twin pairs were allocated in the same batches, which might overestimate the contribution of shared environmental factors. Therefore, random correction for batch effect was carried out.

## Conclusion

Our current findings, along with previous observations, emphasize the significant role of individual circumstances on the settings of the HPA axis. Notably, the contribution of unshared environmental factors on cortisol production was considerable and was found to increase with age, implicating a predominant role of individual circumstances with aging. In contrast to cortisol production, cortisol metabolism was considerably influenced by genetic constitution, and heritability of A-ring reductases was found to increase with age, resulting in a peak of the genetic contribution at the age of 17. For 11β-HSD isozymes and CYP 3A4, this peak was found at the age of 12.

## Additional Information

***Disclosure Summary:*** BRW is a Wellcome Trust investigator.

***Data Availability:*** The datasets generated during and/or analyzed during the current study are not publicly available but are available from the corresponding author on reasonable request.
